# Surface Design for Immobilization of an Antimicrobial Peptide Mimic for Efficient Anti‐Biofouling

**DOI:** 10.1002/chem.202000746

**Published:** 2020-04-21

**Authors:** Abshar Hasan, Kyueui Lee, Kunal Tewari, Lalit M. Pandey, Phillip B. Messersmith, Karen Faulds, Michelle Maclean, King Hang Aaron Lau

**Affiliations:** ^1^ Bio-Interface & Environmental Engineering Lab Department of Biosciences and Bioengineering Indian Institute of Technology Guwahati Assam 781039 India; ^2^ Department of Bioengineering University of California, Berkeley Berkeley USA; ^3^ 1. Department of Bioengineering 2. Department of Materials Science and Engineering University of California, Berkeley Berkeley USA; ^4^ Materials Sciences Division Lawrence Berkeley National Laboratory Berkeley USA; ^5^ 1.Department of Electronic & Electrical Engineering 2.Department of Biomedical Engineering University of Strathclyde 295 Cathedral Street Glasgow G1 1XL UK; ^6^ Department of Pure & Applied Chemistry University of Strathclyde 295 Cathedral Street Glasgow G1 1XL UK

**Keywords:** antimicrobial peptides, bacterial attachment, biointerfaces, click chemistry, peptoids

## Abstract

Microbial surface attachment negatively impacts a wide range of devices from water purification membranes to biomedical implants. Mimics of antimicrobial peptides (AMPs) constituted from poly(*N*‐substituted glycine) „peptoids“ are of great interest as they resist proteolysis and can inhibit a wide spectrum of microbes. We investigate how terminal modification of a peptoid AMP‐mimic and its surface immobilization affect antimicrobial activity. We also demonstrate a convenient surface modification strategy for enabling alkyne–azide „click“ coupling on amino‐functionalized surfaces. Our results verified that the N‐ and C‐terminal peptoid structures are not required for antimicrobial activity. Moreover, our peptoid immobilization density and choice of PEG tether resulted in a „volumetric“ spatial separation between AMPs that, compared to past studies, enabled the highest AMP surface activity relative to bacterial attachment. Our analysis suggests the importance of spatial flexibility for membrane activity and that AMP separation may be a controlling parameter for optimizing surface anti‐biofouling.

## Graphical abstract

**Efficient anti‐biofouling**: We show that an archetypical antimicrobial peptoid could retain high activity even with terminal modifications. Immobilization by alkyne–azide „click“ coupling on one‐step modified amino‐surfaces was demonstrated. Appropriate spatial separation between immobilized peptoids, rather than increasing surface density, was found to promote both resistance against bacterial attachment and AMP surface activity.

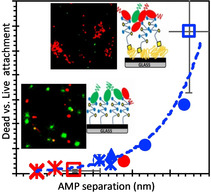

Bacterial adhesion and colonization on implantable biomedical devices and the consequent infection contribute to 40–70 % of hospital‐acquired infections (HAI).^1^ Water purification systems, food packaging, and maritime operations can also be compromised by microbial contamination.^2^ Despite substantial research, prevention of bacterial adhesion and growth on surfaces is still challenging.^3^ Surface properties such as roughness and topology, chemistry and wettability, as well as surface molecular arrangements, are among the many factors that influence biofouling.^4^

Proposed strategies for overcoming bacterial surface fouling include „antifouling“ coatings that inhibit non‐specific protein adsorption and bacterial attachment, such as by surface grafting poly(ethylene glycol) (PEG) as polymer brushes.^5^ Immobilization of existing antibiotics and antibiotic‐releasing coatings are other strategies.^6^ However, many existing antimicrobial agents suffer from a narrow spectrum of activity and a rising resistance against their activities.6b, 7 Antimicrobial peptides (AMPs) are being investigated to overcome these issues,^6^ but they are degraded by proteases secreted by both human hosts and bacteria.^8^

Poly(*N*‐substituted glycine) „peptoids“ represent a promising class of peptidomimics being developed to address the drawbacks of AMPs. They possess a non‐natural polyglycine backbone with sidechains attached to backbone amide nitrogen atoms that offers protease resistance and enhanced lipid membrane permeability.^9^ Secondary structures are induced in specific sequences with specific sidechains.9b, 10

A number of groups have demonstrated peptoid AMP mimics that exhibit high activity.6b, 8a, 11 One such peptoid has also been synthesized as part of a surface grafted peptoid brush but a high level of overall bacterial attachment was observed.^12^ Natural AMPs such as hLf1‐11, LL‐37, and melamine have also been immobilized with varying results.^6^, 8c, 13 These studies apply bioconjugation techniques such as maleimide‐thiol, amide, and alkyne–azide „click“ coupling to enable covalent surface immobilization. Alkyne–azide coupling is especially suitable since it is orthogonal to reactive groups commonly found on AMPs, but the approach is often limited by the availability of specialized chemical linkers.

In the present work, we employ a 12‐mer (Nlys‐Nspe‐Nspe)_4_ antimicrobial peptoid with an amphiphilic helical structure, first reported by Barron et al.,^11^ as a model AMP mimic for investigating the influence of immobilization design on surface antimicrobial activity. We first tested the effects of modifying the peptoid's N‐ and C‐termini with diethylene glycol segments on the minimum inhibitory concentrations (MICs) in solution. We then demonstrated the conversion of surface immobilized amines into azides for copper(I)‐catalyzed alkyne–azide cycloaddition (CuAAC) surface coupling of the peptoid, with or without a 2 kDa polyethylene glycol (PEG) tether. We characterized the surface modification steps by water contact angle (WCA) analysis and X‐ray photoelectron spectroscopy (XPS), and finally assayed the surfaces for protein adsorption and live/dead bacterial attachment. We hypothesized that sufficient spatial separation between AMPs and hence flexibility in molecular arrangement, such as enabled by a PEG tether, is required to both resist bacterial attachment and retain antimicrobial activity on a surface.

The (Nlys‐Nspe‐Nspe)_4_ parent sequence is composed of a repeating „kss“ motif in which k and s are, respectively, the Lys‐analogue *N‐(*4‐aminobutyl)glycine (Nlys) and the α‐chiral (*S*)*‐N‐(*1‐phenylethyl)glycine (Nspe) (Figure 1 A). The sequence represents an archetypical ABB trimer motif in which A is cationic and B is hydrophobic (often Nspe to induce helicity). Peptoid synthesis was carried out using well‐established „sub‐monomer“ solid phase synthesis (SSPS),9b and all sequence modifications were performed on‐resin using commercially available building blocks (see ESI). HPLC and LC‐MS characterization of purified sequences are shown in Figures S1 and S2.

**Figure 1:**
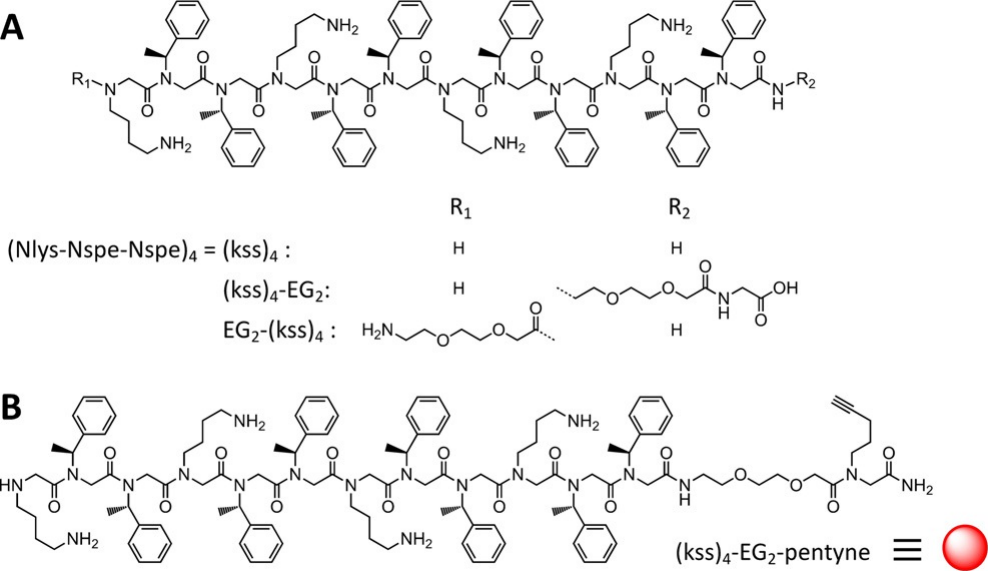
A) Chemical structures of the (kss)_4_ antimicrobial sequence as well as its C‐ and N‐modifications. B) Chemical structure of the modified sequence for CuAAC „click“ coupling. The red ball representation is used in Figure 2 A.

We first verified whether the C‐terminal amide or the N‐terminal amine of (kss)_4_ might be important to its bactericidal effect. Cultured bacteria (5×10^7^ CFU mL^−1^) were incubated in growth broth containing peptoids modified either at the C‐ or N‐terminus with a diethylene glycol (EG_2_) linker to give, respectively (kss)_4_‐EG_2_ and EG_2_‐(kss)_4_ (Figure 1 A). The EG_2_ linker was also used later for spacing (kss)_4_ from the surface‐coupling group (see below). We found similar MICs with or without terminal modifications for the Gram negative and Gram positive strains tested (i.e. 16–20 μm against *Pseudomonas aeruginosa* (PA01), 5–9 μm against *Escherichia coli* (ATCC 25 922), 1–6 μm against *Staphylococcus aureus* (NCTC 4135); see Figure S3 for full data). A previous report modifying the N‐terminus of (kss)_4_ with a small‐molecule metal chelator also showed little change in MIC against E. coli.^14^ A different report modifying the C‐terminus of a peptoid similar to (kss)_4_ with a non‐functional 20‐residue peptoid lowered activity (i.e. increased MIC) by 2–10 times depending on the strain.^12^ The overall data suggest that the peptoid N‐ and C‐terminal structures are not essential to activity, but the steric bulk of the modification may be important.

For surface immobilization, we further modified the C‐terminal with a residue possessing a pentyne sidechain using regular peptoid SSPS to generate (kss)_4_‐EG_2_‐pentyne (Figure 1 B). In parallel, following established protocols (see ESI), we prepared glass slides silanized either with (3‐glycidyloxypropyl)trimethoxysilane (GOPTS) further functionalized by a diamino‐PEG_2k_,^15^ or simply with (3‐aminopropyl)trimethoxysilane (APTMS) (Figure 2 A).^16^ The terminal amines on both surfaces were then converted to azides by one‐step overnight incubation with imidazole‐1‐sulfonyl azide (see ESI).^17^ This enabled CuAAC coupling^18^ of (kss)_4_‐EG_2_‐pentyne to give peptoid functionalized surfaces with and without a PEG_2k_ tether, that is, Figure 2 A Scheme A: GOPTS‐PEG‐N_3_‐(kss)_4_ and Scheme B: APTMS‐N_3_‐(kss)_4_, respectively.

**Figure 2:**
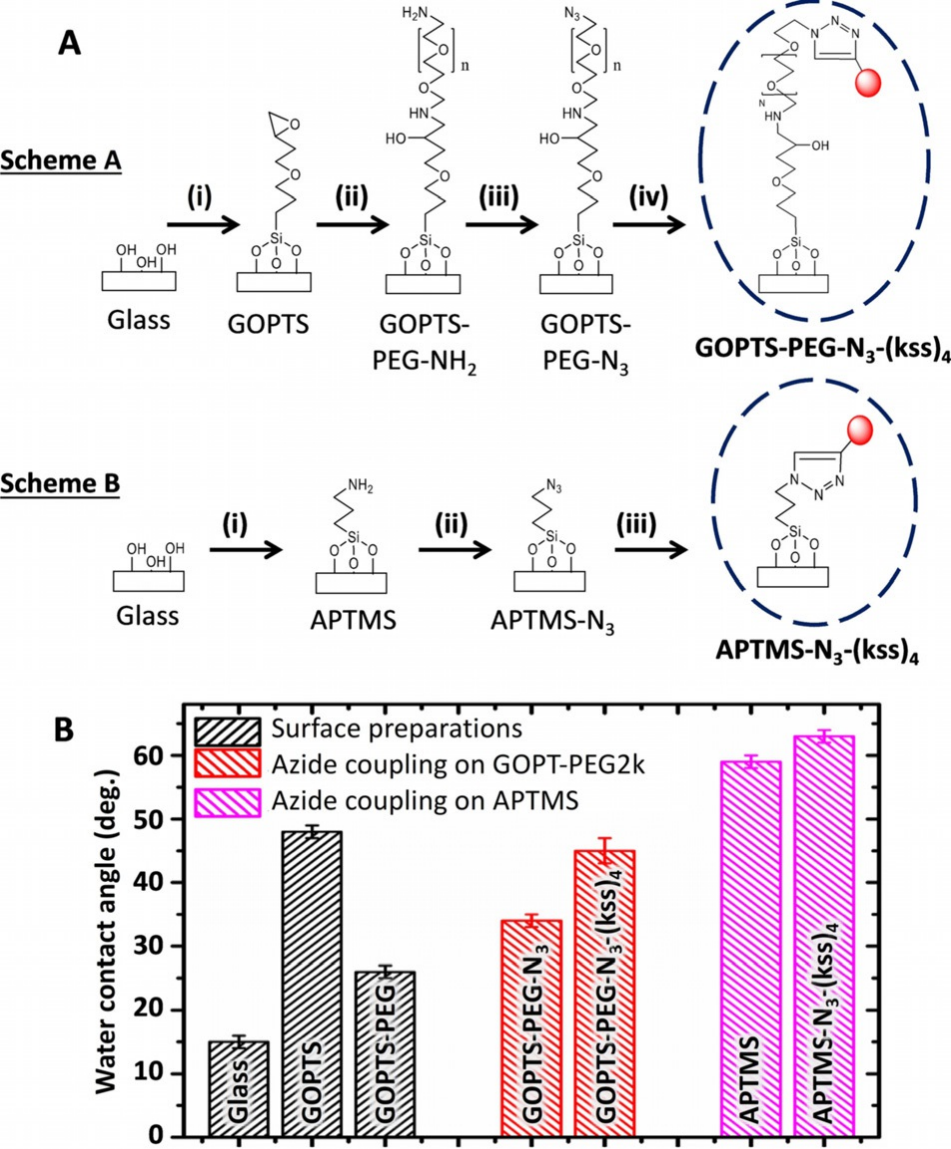
A) Surface modification schemes for generating PEG‐tethered (kss)_4_ (i.e. Scheme A: GOPTS‐PEG‐N_3_‐(kss)_4_) and (kss)_4_ immobilized directly on the surface (i.e. Scheme B: APTMS‐N_3_‐(kss)_4_). B) Water contact angles measured after successive modification steps.

Figure 2 B shows water contact angle (WCA) data consistent with the expected changes in surface wettability after each modification step. For Scheme A, WCA increased with initial GOPTS modification (the organosilane is more hydrophobic than glass), and then decreased after coupling of diamino‐PEG_2K_ (PEG‐amine is hydrophilic). Subsequent conversion of the PEG terminal amine to a non‐cationic azide (N_3_) and then CuAAC coupling of (kss)_4_, which possesses numerous hydrophobic Nspe groups, successively increased WCA. Similarly, for Scheme B, successive increases in WCA was observed after the glass was silanized with the organosilane APTMS and then finally functionalized with (kss)_4_.

The surface modifications were further confirmed by XPS. In the C1s spectrum (Figure 3 A), a peak appeared at 286.5 eV after GOPTS silanization that indicated the expected addition of C−O bonds in the epoxide groups. PEG attachment was verified both by further increases of this C−O peak arising from the abundance of ether bonds in PEG, and by the appearance of the N1s N−C peak (401 eV) arising from the terminal amine of the PEG used (Figure 3 B). Subsequent azide derivatization was confirmed by the appearance of a N−N=N^−^ peak at 402.3 eV.^13^, ^19^ Final peptoid coupling was confirmed by the substantial increase in peaks attributed to (kss)_4_: C1s C−C (284.8 eV) and amide (288.3 eV),^19^ and N1s N−C=O (399.5 eV) and NH_2_ (400.8 eV).^20^ By analyzing the attenuation of the Si2p signal from the SiO_2_ substrate, we estimate a final peptoid surface density of 0.3 chain/nm^2^ (Table S1 and related discussion).

**Figure 3:**
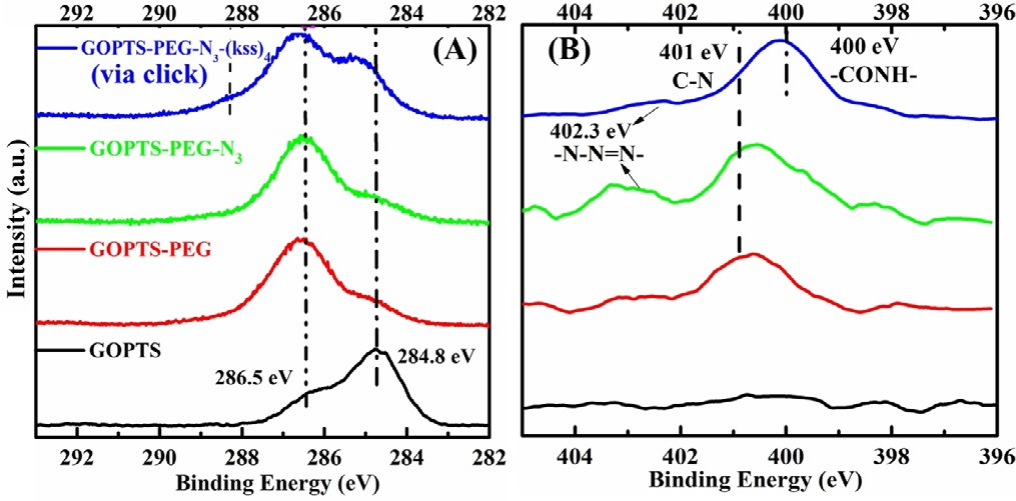
High‐resolution C1s (A) and N1s (B) XPS spectra after each surface modification steps to achieve GOPTS‐PEG‐N_3_‐(kss)_4_.

Our PEG tether essentially forms a polymer brush, which should confer resistance against non‐specific biomolecular adsorption and hence reduce bacterial attachment.5a, 5c For initial evaluation of this anti‐fouling property, we incubated GOPTS‐PEG‐N_3_‐(kss)_4_ samples in 10 % FBS (RT for 2 h). Following established protocol,^21^ ellipsometry measurements showed little change of the adlayer thickness before and after incubation (Figure S4: 3.5±0.6 nm vs. 3.7±0.5 nm, *n*=3), indicating little protein adsorption on the PEG‐tethered peptoid surface.

We then focused on evaluating the antimicrobial activity of the peptoid‐functionalized surfaces against *P. aeruginosa* (PA01) due to its high relevance in HAI and risks associated with biofilm formation.^22^ Figures 4 A–D show typical images of attached live and dead/damaged bacterial cells stained, respectively by Syto 9 and propidium iodide (PI) after a 24 h attachment assay (5×10^7^ CFU mL^−1^, 37 °C). Figure 4 E summarizes this data in terms of actual surface coverage (*θ*_coverage_) and normalized coverage (*θ*_norm_; relative to unmodified glass control).

**Figure 4:**
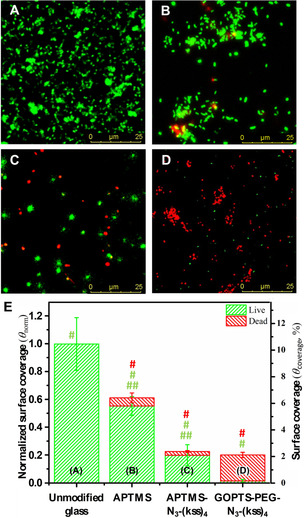
Typical confocal microscopy images of live (green) and dead/damaged (red) *P. aeruginosa* on: A) unmodified glass, B) APTMS, C) APTMS‐N_3_‐(kss)_4_, and D) GOPTS‐PEG‐N_3_‐(kss)_4_. E) Quantified attachment data corresponding to confocal measurements. Both actual coverage (*θ*_coverage_) and coverage normalized to attachment on unmodified glass (*θ*_norm_) are shown. # and ## denote p<0.005 and p<0.05, respectively (one‐way ANOVA).

On unmodified glass, a relatively high live *P. aeruginosa θ*_coverage_=10.5 % (*θ*_norm_≡1) was observed, with only live bacteria found (Figure 4 A). In contrast, on PEG‐tethered (kss)_4_ (i.e. GOPTS‐PEG‐N_3_‐(kss)_4_), a much lower *θ*_norm_=0.21 was observed, of which only a small fraction consisted of live bacteria (*θ*_norm‐live_=0.02) (Figure 4 D and E). In comparison, although a similar overall attachment (*θ*_norm‐total_=0.23) was observed on (kss)_4_ immobilized without PEG (i.e. APTMS‐N_3_‐(kss)_4_), most of these cells were still live (*θ*_norm‐live_=0.20) (Figure 4 C and E). Therefore, the 2 kDa PEG tether was instrumental to achieving high surface activity.

We performed a further control with APTMS modified glass, which gave an amine‐terminated surface (Figure 2 A, Scheme B, step [i]), to mimic the positive charge expected on (kss)_4_ surfaces. Figures 4 B and E show *θ*_coverage_=6.5 % on APTMS, consisting mostly of live bacteria. This level of attachment was moderately lower than the control (*θ*_norm_=0.62), a phenomenon that has occasionally been observed on amino‐silane surfaces (Figure S5).^23^ However, this was still about 3‐times higher than on the (kss)_4_ functionalized surfaces. This suggests a role of the antimicrobial sequence in suppressing attachment, notwithstanding its cationic nature, that might be related to the ability of similarly short surface‐grafted peptoids in resisting biofouling.21a, 21b As for the minor fraction of dead/damaged attached cells (*θ*_norm‐dead_=0.06), a role for electrostatic surface adhesion that compromises the fluidity and integrity of bacterial membranes could be possible.

We had also performed our attachment assay against *E. coli* (ATCC 25922) but only very little attachment was observed and no statistically significant data were obtained. It is possible that some detachment had occurred under our conditions. Nonetheless, based on the even lower MIC measured for our modified peptoids against *E. coli* (and *S. aureus*) than *P. aeruginosa* (Figure S3), we anticipate that GOPTS‐PEG‐N_3_‐(kss)_4_ surface modification would be effective against these strains.

Overall, the results for our PEG‐tethered peptoid were characterized by low live bacterial attachment and a high proportion of dead/damaged cells. Our immobilized (lateral) density of 0.3 chain/nm^2^ (see XPS analysis), considered together with the flexibility in both lateral and vertical movement allowed by the 20 nm contour length of PEG‐N_3_‐(kss)_4_, imply a maximum „volumetric“ separation of about 5 nm between immobilized (kss)_4_ sequences (see ESI for calculations). This is equivalent to the average molecular separation found in a 25 mm solution, which is orders of magnitude higher than the MICs of (kss)_4_. Thus, surface immobilization can generate a very high local concentration of AMPs.

Indeed, past studies have focused on increasing the immobilized density of AMPs.^12^, ^23^, ^24^, ^25^ However, AMPs generally possess hydrophobic and cationic groups, both of which promote undesirable bacterial attachment. Plotting our results alongside past studies, where data for calculating AMP separation are available (see ESI), shows many reports of high live attachments, especially those with relatively shorter AMP separations (i.e. high AMP densities) (Figure 5 A). Immobilization directly on silanized surfaces generally resulted in the shortest separations since silanization gives a high density of surface coupling groups. Tethering AMPs at the tip of polymer brushes, including our design, generally increased separations because the polymer chains prevent close packing and enable lateral and vertical movement around the anchor point of the polymer tether. However, some studies had attached multiple AMPs along the length of the polymer chains to increase immobilization density,23b, 24 reducing AMP separation. Overall, Figure 5 A shows it is possible to decrease live attachment by increasing AMP separation, despite the diverse bacteria types and assay protocols surveyed. Moreover, our current design coupling a single AMP on PEG_2k_ gave the lowest attachment at the largest AMP separation. This lowered fouling was corroborated by the low FBS adsorption observed (Figure S6).

**Figure 5:**
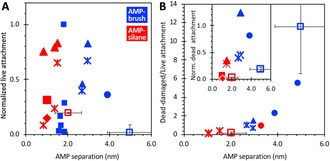
A) Live bacterial attachment normalized to levels on unmodified substrate (glass or Ti). B) Ratio of dead/damaged bacterial attachment versus live attachment shown in (A). The inset shows the original dead attachment data. Open squares (□) indicate the present study for *P. aeruginosa*. Other symbols indicate literature data for *P. aeruginosa* (▪),23c, 24 *E. coli* (•),^12^, ^25^ *S. aureus* (⧫),23c *L. salivarius* (▴),23a, 23b and *S. sanguinis* (✶).23a, 23b Attachment was measured by either imaging stained cells or re‐culturing of attached bacteria.

Turning to damaged/dead bacterial attachment, Figure 5 B‐inset shows that AMPs coupled at intermediate (3–4 nm) separations on brushes exhibited the highest apparent surface activity (i.e. highest dead attachments). This is consistent with our hypothesis that a polymer tether can introduce flexibility in molecular arrangement and orientation for enhanced membrane interactions. However, attached dead bacteria could still lead to biofilm formation as well as acute immune responses. Figure 5 B plots the same data ratioed against live attachment, to highlight cases with low overall attachment as well as relatively high activity. This reveals a remarkable correlation between increasing AMP separation and relative activity, despite the diverse experiments compared. In fact, whereas our APTMS‐N_3_‐(kss)_4_ design exhibited a low relative activity similar to other silane surfaces, our GOPTS‐PEG‐N_3_‐(kss)_4_ brush design had the highest separation (5 nm) as well as the highest relative activity. Naturally, it can also be expected that the relative activity would decrease at very large AMP separations, which implies a very low density of AMPs insufficient for disrupting the membrane of a bacterium. An intermediate AMP separation should therefore exist for exhibiting an optimal relative activity.

In conclusion, we have shown that a model antimicrobial peptoid AMP mimic is amenable to modification of both its C‐ and N‐ termini, and we demonstrated a one‐step protocol for introducing azide‐terminations on amino‐functionalized surfaces for CuAAC „click“ surface coupling. These demonstrations enabled a study of AMP immobilization design showing that surface activity is strongly enhanced by a polymer (PEG_2k_) tether, consistent with the importance of engineering spatial flexibility and vertical reach for suitable surface interactions with bacteria. Moreover, we introduce AMP separation as a new parameter for characterizing immobilized AMP anti‐biofouling. This parameter highlights the very high local AMP concentrations achieved by surface immobilization. It also reveals, by comparison with literature data, a strong correlation between increasing AMP separation and increasing relative surface activity, indicated by a high proportion of dead/damaged bacteria among a low level of attachment. In fact, our PEG coupling design exhibited the largest AMP separation and also the highest relative activity. The present results therefore highlight the potential of optimizing AMP separation, rather than immobilization density, to enable both surface activity and reduced bacterial attachment.

## Conflict of interest

The authors declare no conflict of interest.

## Supporting information

As a service to our authors and readers, this journal provides supporting information supplied by the authors. Such materials are peer reviewed and may be re‐organized for online delivery, but are not copy‐edited or typeset. Technical support issues arising from supporting information (other than missing files) should be addressed to the authors.

Supplementary
