# Supplementary material for: Surface Design for Immobilization of an Antimicrobial Peptide Mimic for Efficient Anti‐Biofouling
Source: Chemistry. 2020 Apr 21;26(26):5789–93. doi: 10.1002/chem.202000746 (PMC7318250; doi:10.1002/chem.202000746)
Supplement: Supplementary file 1 — Supplementary [file CHEM-26-5789-s001.pdf]

Chemistry–A European Journal

Supporting Information

Surface Design for Immobilization of an Antimicrobial Peptide Mimic for Efficient Anti-Biofouling

Abshar Hasan,[a, f] Kyueui Lee,[b] Kunal Tewari,[f] Lalit M. Pandey,[a] Phillip B. Messersmith,[c, d] Karen Faulds,[f] Michelle Maclean,[e] and King Hang Aaron Lau*[f]

Author Contributions

A.H. Conceptualization: Supporting; Data curation: Lead; Formal analysis: Lead; Funding acquisition: Lead; Investigation: Lead; Methodology: Lead; Writing - Original Draft: Lead
K.L. Formal analysis: Supporting; Investigation: Supporting; Writing - Review & Editing: Supporting
K.T. Investigation: Supporting
L.P. Supervision: Supporting
P.M. Supervision: Supporting; Writing - Review & Editing: Supporting
K.F. Funding acquisition: Supporting; Resources: Supporting; Writing - Review & Editing: Supporting
M.M. Investigation: Supporting; Methodology: Supporting; Resources: Supporting; Supervision: Supporting; Writing - Review & Editing: Supporting
K.L. Conceptualization: Lead; Data curation: Supporting; Formal analysis: Supporting; Investigation: Supporting; Resources: Lead; Supervision: Lead; Writing - Review & Editing: Lead.

1. Material and methods: 2
1.1. Materials 2
1.2. Peptoid Synthesis and Surface Immobilization 2
1.2.1. Peptoid Synthesis, Purification and Characterization: 2
1.2.2. Peptoid surface preparation: 3
1.3. Surface characterization 4
1.3.1. Water contact angle (WCA) analysis: 4
1.3.2. X-ray photoelectron spectroscopy (XPS) analysis: 4
1.3.3. Ellipsometry analysis: 4
1.3.4. Protein adsorption 4
1.4. Antibacterial assay 5
1.4.1. MIC assay in solution: 5
1.4.2. Live/dead assay on modified surface: 5
2. Supplementary data 6
2.1. Analytical HPLC of purified peptoid sequences. 6
2.2. LCMS of purified fractions of $(kss)_4$ and modified $(kss)_4$ sequences. 6

2.3. MIC analysis of $(kss)_4$ and modified sequences. 7
2.4. XPS data. 7
2.4.1. XPS data for Scheme A surface modification. 7
2.4.2. Calculation of layer thickness and chain density 7
2.5. Calculation of average AMP separation on surfaces: 8
2.6. Summary of current and published data on immobilized AMPs 10
2.7. Ellipsometry data. 12
2.7.1. FBS adsorption. 12
2.7.2. Lower bound estimation of $(kss)_4$ AMP separation. 12
2.8. Comparison of live bacteria attachment on amino-silanes 13
3. References: 13

1. Material and methods:

1.1. Materials

N-methylpyrrolidone (NMP) and peptide grade dimethyl-formamide (DMF) were procured from Rathburn Chemicals (Walkerburn, Scotland). Acetonitrile of HPLC grade and other solvents of ACS grade (diethyl ether, dichloromethane, 2-Propanol, and methanol), 4-Pentyn-1-amine, N,N-Diisopropylethylamine (DIPEA), piperidine, bromoacetic acid (BAA), trifluoroacetic acid (TFA), and triisopropylsilane (TIPS), (3-glycidyloxypropyl)trimethoxysilane (GOPTS), and (3-aminopropyl)trimethoxysilane (APTMS) were purchased from Sigma-Aldrich, UK. N,N-Diisopropylcarbodiimide was purchased from Alfa Aesar. Tert-butyl N-(4-aminobutyl)carbamate (NLys) and (1S)-1-Phenylethylamine (Nspe) monomers were purchased from Apollo scientific, UK. Hexafluorophosphate Benzotriazole Tetramethyl Uronium (HBTU) and Fmoc-Amino-3,6 dioxaoctanoic acid were purchased from Novabiochem and FluroChem (UK), respectively. Rink amide MBHA (Cat no. 855003) and H-Gly-2-ClTrt (Cat no. 856053) resin were procured from Merck, UK. Round glass coverslips of 14 mm diameter were purchased from VWR.

1.2. Peptoid Synthesis and Surface Immobilization

1.2.1. Peptoid Synthesis, Purification, and Characterization:

Peptoid synthesis was carried out manually using the established submonomer solid phase synthesis (SSPS) technique.[1-3] Briefly, resin was initially swollen in DCM followed by DMF. The Fmoc protection group originally on the as-bought resin was then removed using 20% piperidine in DMF (two times for 20 min each; the reaction vessel with resin was shaken with a rotary shaker at room temperature). Afterwards, bromoacetylation was performed on the terminal amine groups present on deprotected resin by the addition of BAA (20x equivalence of resin) with DIC (18.5x equivalence) and shaken for 15 min at room temperature. Then, the desired sidechain was attached by adding the appropriate primary amine submonomer through S_N2 displacement on the terminal bromide (20x excess, 1M in NMP; 30 min under shaking at

room temperature). The bromoacetylation and S_N2 displacement steps were repeated for each additional residue attached. The primary amines used were: tert-butyl N-(4-aminobutyl)carbamate (NLys), (1S)-1-Phenylethylamine (Nspe), and 4-pentyn-1-amine for the residue with the alkyne sidechain.

For incorporating the EG_2 unit, a commercially available Fmoc-Amino-3,6 dioxaoctanoic acid monomer was coupled to the N-terminus on the resin using conventional peptide solid phase synthesis protocol with HBTU as the coupling agent. Fmoc-amino-3,6 dioxaoctanoic acid and HBTU were together dissolved in DMF (both 6x equivalence) with DIPEA added (9x equivalence). The mixture was added to the resin and shaken for 2 h at 37°C. To ensure complete coupling, the procedure was repeated (i.e. double coupling).

Rink amide MBHA resin was used to give an amide C-terminus for all sequences, except for the test sequence $(kss)_4$-EG_2 i.e. EG_2 on the C terminus. In this case a H-Gly-2-ClTrt resin was used to give a C-acid terminal Gly residue.

Post synthesis, the peptoid chains were cleaved by treating rink amide resin with a TFA cocktail typically used in solid phase synthesis (95:2.5:2.5 TFA:TIPS:H_2O v/v/v) for 30 min. The TFA filtrate was dried in a rotary evaporator and the peptoid was precipitated with diethyl ether. The precipitate was collected, freeze dried, and finally purified using preparative gradient RP-HPLC (Dionex Ultimate 3000) using a 250x10 mm Phenomenex Jupiter C18 column.

Fractions containing the product were identified by ESI-LC-MS analysis (Agilent 1200 with a Poroshell C18 column coupled to an Agilent 6130 Mass Spectrometer) and their purities were characterized by analytical RP-HPLC (Dionex P680) using a 250x4.6 mm "Nucleosil" C18 column (Macherey-Nagel). A 1 mL/min flow rate and 30 min gradient of 5−95% acetonitrile in water containing 0.1% TFA was used. High purity fractions were freeze dried and stored at -25°C for later use. Example mass spectra and HPLC chromatograms of the pure fractions are shown in Figures S1 and S2.

1.2.2. Peptoid surface preparation:

Glass coverslips were cleaned by washing with plenty of water, then sonication in 1% Hellmanex reagent for 20 min, followed by washing with water and a final sonication for 5 min. Washed surfaces were dried using N_2 and further cleaned in O_2 plasma cleaner (Femto plasma systems, Diener, Germany). Surfaces were silanized with either GOPTS (for Scheme A, Fig.2) or APTMS (for Scheme B, Fig.2) by following a previously established gas phase deposition in a vacuum oven at 100°C for 1 h.[4]

To attach a PEG2k tether on GOPTS, the silanized samples were further modified by coupling diamino-PEG (Creative PEGworks, US. Molecular weight: 2 kDa) by referring to a previously reported method established by Ulijn et al.[5] Briefly, 10 $mg.mL^{-1}$ of diamino-PEG solution was prepared in methanol, drop casted on silanized surfaces and dried at 40°C in vacuum for 1 h to evaporate the solvent. The temperature was then raised to 100°C and left to react for 24 h in vacuum. Post incubation, surfaces were thoroughly washed to remove unreacted PEG molecules and again dried under vacuum.

To obtain azido groups on both the APTMS and amino-PEG functionalized surfaces, and thereafter couple the (kss)$_4$-EG2-pentyne peptoid using azide-alkyne click chemistry, the samples were further modified by applying existing protocols originally developed for the solution phase.[6, 7] The samples with free primary amines were first dipped in a H_2O/methanol (1:2, 10 mL) mixture. A solution of imidazole-1-sulfonyl azide·HCl (17.9 mmol), $NaHCO_3$ (96.54 mmol) and $CuSO_4.5H_2O$ (0.149 mmol) in water was added to the samples immersed in H_2O/methanol, and the entire mixture was stirred for 16 h at room temperature.[7] Post incubation, the surfaces were washed with water, dried under N_2 and examined for azide formation using WCA and XPS. The last step of surface modification was the coupling of peptoid to the surface via Cu(I)-catalyzed azide–alkyne cycloaddition reaction (CuAAC) with slight modifications.[6, 8] Briefly, 3 to 5 azide modified surfaces were dipped in 3 mL of alkyne-peptoid solution (1 mg.mL^{-1} in DMSO) in the presence of $CuSO_4$ (1M, 12 µL) and sodium ascorbate solution (45 mg in 125 µL). The surfaces were left on continuous stirring overnight. After incubation, surfaces were thoroughly washed with water and dried under N_2.

1.3. Surface characterization

1.3.1. Water contact angle (WCA) analysis:

Goniometer DSA25 (Krüss) was used to measure the static WCA using the sessile drop method.[9, 10] Water drops of volume 3 µL were dropped with the help of automated dispensing system and images of the drop profile were captured after 10 s. Contact angles at the solid-liquid interface were determined by circular fitting of the drop outline using in-built Krüss software. At least three measurements on two different samples for each type of surface were recorded.

1.3.2. X-ray photoelectron spectroscopy (XPS) analysis:

XPS analysis of functionalized substrates in each step was performed using Phi 5600 XPS (Perkin Elmer, U.S.). Survey and high-resolution C1s and N1s spectra were measured via a monochromatic Al Kα X-ray source operated at 350 W. To prevent surface charging of the functionalized substrates, a neutralizer with a constant current of 1 µA was simultaneously used during the measurement. MATLAB-based MultiPak software developed by Physical Electronics, Inc. (PHI, U.S.) was used to identify the atomic percentages of each element from survey spectra. Deconvolution of the high-resolution C1s and N1s spectra was done in OriginPro 8.5 software.

1.3.3. Ellipsometry analysis:

The thicknesses of the peptoid-immobilized SiO_2 surfaces before and after the incubation in 10% fetal bovine serum (FBS) solution (RT, 2 h) were measured using M44 spectroscopic ellipsometer (J.A. Woollam, U.S.). Each measurement was carried out under three different angles: 55, 60, and 65° in the wavelength range 300 to 900 nm. The experimental data sets were fit using Cauchy optical model in CompleteEase software developed by J.A. Woollam. Experiments were performed in triplicates (n = 3) to calculate standard deviation.

1.3.4. Protein adsorption

Peptoid modified surfaces were tested for protein adsorption following an established methodology.[2, 11, 12] Briefly, surfaces were incubated in 10% FBS solution in phosphate buffer

saline (PBS) at room temperature for 2 h. Post incubation surfaces were washed thoroughly with PBS and then by deionized (DI) water to remove salt particles from the surface and later dried under N_2 for ellipsometry characterization.

1.4. Antibacterial assay

Escherichia coli (ATCC 25922), *Pseudomonas aeruginosa* (PA01), and *Staphylococcus aureus* (NCTC 4135) strains were used for determining the antibacterial properties of the peptoids. All bacterial strains were grown overnight at 37°C in Luria-Bertani (LB) broth (Sigma-UK).

1.4.1. MIC assay in solution:

Broth microdilution method (CLSI M07-A10) was employed for determining minimal inhibition concentrations (MIC) and was performed in Mueller–Hinton (MH) broth using bacterial culture grown until logarithmic phase. Briefly, peptoids dissolved in MH broth at required concentrations were added to 96 well microtiter plates at a bacterial inoculum concentration of $5x10^7$ CFU/mL. MH broth with the same inoculum concentration and without peptoid served as control. Plates were then incubated at 37°C overnight (~16 h). Experiments were performed in triplicates (n = 3).

1.4.2. Live/dead assay on modified surface:

Antimicrobial testing of peptoid modified surfaces was performed using fluorescence imaging based live/dead analysis using FilmTracer Live/Dead biofilm viability kit (Cat No. L10316, Invitrogen). Briefly, modified circular glass coverslips surfaces (14 mm diameter) were initially UV sterilized for 30 min and placed in 24 well plates and then incubated with 1 mL of bacterial inoculum ($5x10^7$ CFU/mL of PA01 *P. aeruginosa* or ATCC 25922 *E. coli*) prepared in sterile 0.9% NaCl solution. Surfaces were left for incubation at 37°C for 24 h.

Post incubation, the bacterial suspension was carefully decanted and surfaces were transferred to fresh wells and rinsed with saline solution just once. Further, 300 µL of BacLight live/dead Syto 9/PI staining solution (prepared as per manufacturer's protocol) was dropped on surfaces and left for incubation for 30 min. The red fluorescent PI penetrates only bacteria with damaged membrane and reveals "dead" cells. The green Syto 9 stains for "live" cells—it penetrates all cell membranes but its fluorescence is quenched by PI in dead/damaged cells. After staining, surfaces were rinsed twice gently with MilliQ water and imaged using a confocal microscope (Leica SP8, Germany).

2. Supplementary data

2.1. Analytical HPLC of purified peptoid sequences.

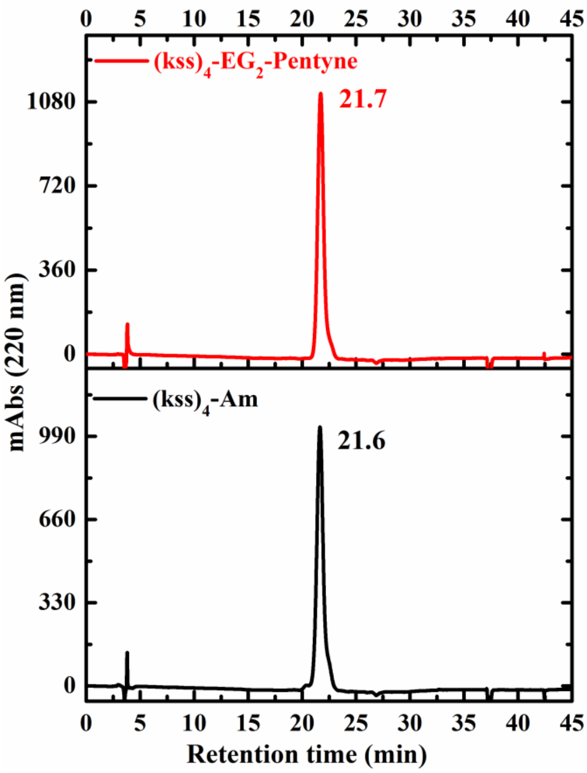

Figure S1. (A) RP-HPLC chromatogram of purified fractions of $(kss)_4$-Am) and $(kss)_4$-EG_2-Pentyne sequence. ACN gradient of 5-95% was used over a period of 30 min.

2.2. LCMS of purified fractions of $(kss)_4$ and modified $(kss)_4$ sequences.

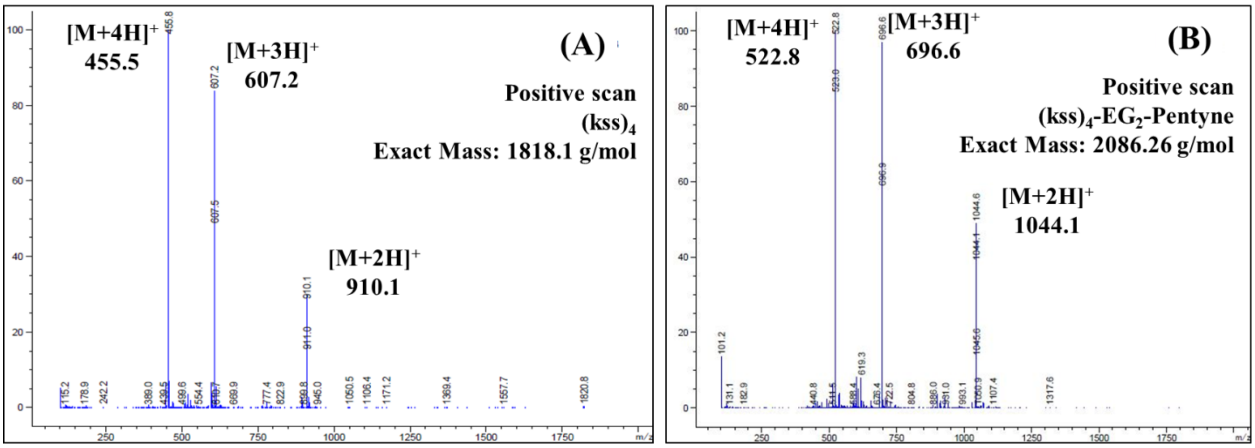

Figure S2. LCMS spectra (counts vs m/z) exhibiting positive ion mode for $(kss)_4$ (A) and $(kss)_4$-EG_2-Pentyne (B) peptoid fractions.

2.3. MIC analysis of $(kss)_4$ and modified sequences.

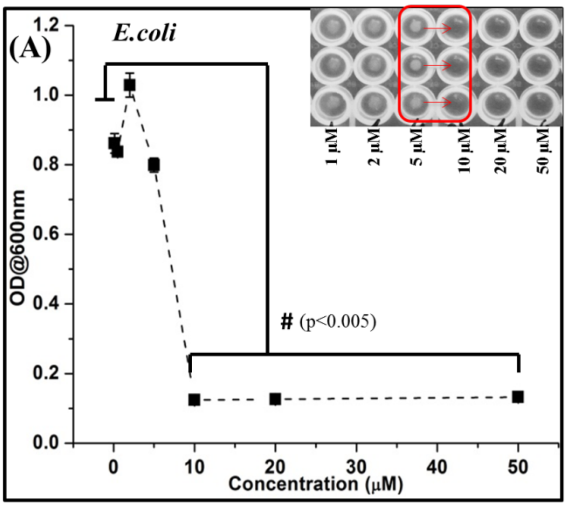

(B)

Sample	$(kss)_4$	$(kss)_4$-EG_2	EG_2-$(kss)_4$
**Molecular weight**	1819.4	2021.1	1963.1
**HPLC elution (% ACN)**	64.8	64.2	64.2
***MIC, E. coli* (ATCC 25922) (μM)**	5.3	8.5	5.7
***MIC, P. aeruginosa* (PA01) (μM)**	15.6	18.7	20.0
***MIC, S. aureus* (μM)**	1.6	5.6	1.2

Figure S3. MIC analysis of peptoid 1 and C terminal modified peptoid 1 sequences. (A) Shows fitting of representative MIC data for Peptoid 1. Inset shows the image of 96 well plate from broth microdilution assay. (B) Table comprising of molecular weight, HPLC elution and MIC (broth microdilution assay) of peptoids. MIC was tested against *E. coli* (ATCC 25922), *P. aeruginosa* (PA01) and *S. aureus* strains (NCTC 4135). MIC experiment was triplicated to determine the standard deviation. MIC values represented in table are the fitted values calculated using Exponential decay function.

2.4. XPS data.

2.4.1. XPS data for Scheme A surface modification.

Table S1. XPS elemental composition of the modified surfaces at each step of modification.

Surface	C1s %	O1s%	N1s%	Si2p%
GOPTS	17.9	54.4	-	21.9
GOPTS-PEG	33.6	44.8	1.3	14.5
GOPTS-PEG-N_3	16.4	55.7	small	21.9
GOPTS-PEG-N_3-$(kss)_4$ (via click)	28.1	48.3	1.8	17.3
XPS sensitivity factor	0.296	0.477	0.711	0.283

2.4.2. Calculation of layer thickness and chain density

The atomic compositions (n_i) shown in Table S1 are related to the signal intensities I_i by the XPS sensitivity factors S_i according to:

$$\frac{n_1}{\sum_{i=0,1,2\ldots} n_i} = \frac{I_1/S_1}{\sum_{i=0,1,2\ldots} I_i/S_i}$$

The change in layer thickness from the coupling of $(kss)_4$ is calculated from the attenuation of an XPS signal unique to the glass substrate (based on Si2p):

$$\frac{I}{I_0} = \frac{e^{-h/\lambda}}{e^{-0/\lambda}} = e^{-d/\lambda} = \frac{n_{(Si)}}{n_{0(Si)}}$$

where I_0 and I are the signal intensities before and after coupling, h is the thickness of the $(kss)_4$ layer, λ is the characteristic attenuation length of Si2p photoelectrons.

Taking a typical value of λ_{Si2p} = 4 nm through an organic layer, the additional layer thickness of $(kss)_4$ immobilized on GOPTS-PEG is calculated:

$$\frac{I}{I_0} = \frac{e^{-h/\lambda}}{e^{-0/\lambda}} = e^{-d/\lambda} = \frac{n_{(Si)}[\text{GOPTS-PEG-N}_3\text{-(kss)}_4]}{n_{0(Si)}[\text{GOPTS-PEG-pentyne}]} = \frac{17.3}{21.9}$$

$$d = -(4\text{ nm}) \ln\left(\frac{17.3}{21.9}\right) = 0.7\text{ nm}$$

From the peptide layer thickness, the surface mass density can be calculated, given the peptide volumetric mass density is known (1.5 g/cm^3)[12]:

$$\text{surface mass density [mass/area]} = \text{thickness [length]} \times \text{volumetric density [mass/volume]}$$

$$= 0.7\, nm \times 10^{-7}\, \frac{cm}{nm} \times 1.5\, \frac{g}{cm^3} \times 10^9 \frac{ng}{g} = 106 \frac{ng}{cm^2}$$

From the surface mass density, the surface chain density can also be calculated:

$$\text{surface chain density} \left[\frac{\text{chains}}{\text{area}}\right] = \text{Avogadro's number} \times \frac{\text{surface mass per area [g/area]}}{\text{molecular weight [g/mole]}}$$

$$= 6.022 \times 10^{23} \times \frac{106 \frac{ng}{cm^2} \times 10^{-14}\, \frac{cm^2}{nm^2}}{2086\, \frac{g}{mole} \times 10^9\, \frac{ng}{g}} = 0.306\, nm^2$$

2.5. Calculation of average AMP separation on surfaces:

For AMPs tethered on a polymer brush, the average separation in 3-D space is based on the "accessible volume" per AMP chain (*V*), which is different from the conventionally reported grafted (2-D) surface chain density of the brush. The grafted density informs the amount of AMP immobilized on the surface but does not take into account the degree of freedom that AMPs may or may not have for interacting with a bacterial membrane once it is in contact with the surface. The AMP may be overly constrained at one end if the polymer brush/linker tether is too short, or the density of AMPs may be so high that multiple AMPs are effectively overlapping each other, thereby either limiting the interaction of each individual sequence or contributing to a hydrophobic surface that promotes irreversible bacterial attachment.

We estimate the accessible volume of a single brush chain from its contour length (contributed by the polymer and the AMP sequence itself), multiplied by the average area taken up by each chain (i.e. inverse of the chain density) – in other words multiplying the chain's maximum

length with its average footprint, thus giving a volume in which the AMP tethered on a chain may potentially access. The average separation, *d*, is calculated by idealizing that *V* is in fact a spherical volume and the separation its diameter, i.e. $V = (2/3)\pi d^3$. Polymer chains generally do not stretch out to their full contour length due to random thermal conformational fluctuations. Therefore, the calculated chain separation is an upper bound estimate.

The contour length is estimated from the per monomer length of peptoids (0.35 nm), peptides (0.34 nm), PEG (0.3 nm), and vinyl polymers (0.25 nm). Accordingly, given the presence of 13 peptoid residues, 45 EG monomers in PEG2k plus 2 in GOPTS-PEG2k-pentyne, and 1.2 nm estimated for the pentyne-azide linker segment, the contour length of GOPTS-PEG2k-N_3-$(kss)_4$ was estimated to be 20 nm.

In different studies, the grafted surface density is reported either directly or in terms of moles per area (e.g. moles/cm^2), immobilized mass per area (e.g. ng/cm^2) or dry layer thickness (in nm). For our present data, the conversion to surface density from thickness and mass per area is described in detail above (section 2.4.2). Thus, the accessible volume for our $(kss)_4$ immobilized on the PEG2k design is:

$$\text{volume per chain} = \text{contour length [length]} \times 1/\text{surface chain density} \left[\frac{\text{chains}}{\text{area}}\right]$$

$$= 20\,nm/0.306\,nm^{-2} = 65\,nm^3$$

and the averaged separation between $(kss)_4$, *d*, is:

$$d = \sqrt[3]{\frac{6V}{\pi}} = \sqrt[3]{\frac{6 \times 65\,nm^3}{\pi}} = 5\ \text{nm}$$

From moles per area, the surface density in chains/nm^2 is given by multiplying moles by the Avogadro's number and by appropriate unit conversions to nm^2.

The volumetric density of PEG is ca. 1.3 g/cm^3 and is estimated to be 1.5 g/cm^3 for peptoids, as described in previous publications.[2, 11] A value of 1.5 g/cm^3 is also used for peptides and other polymers encountered in the literature. The calculated AMP separations are given in Table S2 below.

For multiple AMPs bound on a single polymer chain, *d* is calculated from the contour length of the entire polymer. This then gives the upper bound of the potential flexibility offered by the brush architecture.

For AMPs immobilized directly on the terminal group of a silane (i.e. a very short tether that acts to restrain the AMP at one end to the surface), the separation is calculated directly from the AMP surface chain density. The average area per chain (*A*) is the inverse of the chain density, and the separation is taken as the diameter (*d*) of a circular area matching this per chain area, i.e. $A = \pi d^2/4$.

2.6. Summary of current and published data on immobilized AMPs

Table S2. Live and dead/damaged bacteria attachment reported in the current and past studies.

Reference and surface preparation	AMP separation (nm)	Bacterial species	Norm. live attachment	Norm. dead/damaged attachment	Dead/damaged live ratio
Present work					
Unmodified glass	n/a	*P. aeruginosa*	1.00	0.00	0.00
GOPTS-N3-PEG-kss4	4.69	*P. aeruginosa*	0.02	0.19	11.59
APTMS-N3-kss4	1.86	*P. aeruginosa*	0.20	0.03	0.13
APTMS	n/a	*P. aeruginosa*	0.55	0.06	0.11
[a] Gao et al., 2011					
Ti/TiO2	n/a	*P. aeruginosa*	1.00	n/a	n/a
APTES-PDMA	n/a	*P. aeruginosa*	1.00	n/a	n/a
APTES-PDMA-Tet-213	1.65	*P. aeruginosa*	0.16	n/a	n/a
APTES-PDMA-1010cys	1.68	*P. aeruginosa*	0.08	n/a	n/a
APTES-PDMA-Tet-20	1.87	*P. aeruginosa*	0.02	n/a	n/a
APTES-PDMA-Tet-21	1.82	*P. aeruginosa*	1.00	n/a	n/a
APTES-PDMA-Tet-26	1.86	*P. aeruginosa*	0.28	n/a	n/a
APTES-PDMA-MXX226	1.58	*P. aeruginosa*	0.03	n/a	n/a
[b] Chen et al., 2016					
Ti/TiO2	n/a	*P. aeruginosa*	0.88	0.12	0.13
APTES-mal-melimine	1.04	*P. aeruginosa*	0.31	0.06	0.19
APTES-mal	n/a	*P. aeruginosa*	1.03	0.13	0.12
[b] Chen et al., 2016					
Ti/TiO2	n/a	*S. aureus*	0.91	0.09	0.10
APTES-mal-melimine	1.04	*S. aureus*	0.14	0.02	0.11
APTES-mal	n/a	*S. aureus*	1.88	0.09	0.05
[c] Statz et al., 2008					
Ti/TiO2	n/a	*E. coli*	0.95	0.05	0.05
AMP-PMP1-20	3.89	*E. coli*	0.36	0.81	2.23
Control sequence on PMP1-20	3.89	*E. coli*	2.54	0.01	0.00
PMP1-20	3.89	*E. coli*	0.20	0.07	0.37
[d] Gabriel et al., 2006					
Ti/TiO2	n/a	*E. coli*	n/a	n/a	1.00
APTES-mal-PEG-LL37	4.88	*E. coli*	n/a	n/a	5.50
APTES-mal-LL37	3.33	*E. coli*	n/a	n/a	0.90

[e] Godoy-Gallardo et al., 2014					
Ti/TiO2	n/a	*S. sanguinis*	1.00	n/a	n/a
CPTES-hLf1-11	0.84	*S. sanguinis*	0.08	n/a	n/a
CPTES	n/a	*S. sanguinis*	0.68	n/a	n/a
APTES-mal-hLf1-11	1.38	*S. sanguinis*	0.24	n/a	n/a
APTES	n/a	*S. sanguinis*	0.62	n/a	n/a
[f] Godoy-Gallardo et al., 2015					
Ti/TIO2	n/a	*S. sanguinis*	0.97	0.03	0.03
BUTCS-PDMA-hLf1-11	2.68	*S. sanguinis*	0.46	0.46	1.00
APTES-PDMA-hLf1-12	2.96	*S. sanguinis*	0.67	0.45	0.67
APTES-hLf1-12	1.51	*S. sanguinis*	0.65	0.28	0.43
APTES	n/a	*S. sanguinis*	0.85	n/a	n/a
BUTCS-PDMA	n/a	*S. sanguinis*	0.74	n/a	n/a
APTES-PDMA	n/a	*S. sanguinis*	0.91	n/a	n/a
[e] Godoy-Gallardo et al., 2014					
Ti/TiO2	n/a	*L. salivarius*	1.00	n/a	n/a
CPTES-hLf1-11	0.84	*L. salivarius*	0.75	n/a	n/a
CPTES	n/a	*L. salivarius*	0.98	n/a	n/a
APTES-hLf1-11	1.38	*L. salivarius*	0.79	n/a	n/a
APTES	n/a	*L. salivarius*	1.01	n/a	n/a
[f] Godoy-Gallardo et al., 2015					
Ti/TIO2	n/a	*L. salivarius*	1.00	0.00	0.00
BUTCS-PDMA-hLf1-11	2.68	*L. salivarius*	0.39	0.39	1.00
APTES-PDMA-hLf1-12	2.96	*L. salivarius*	0.83	1.24	1.50
APTES-hLf1-12	1.51	*L. salivarius*	0.83	0.36	0.43
APTES	n/a	*L. salivarius*	0.84	n/a	n/a
BUTCS-PDMA	n/a	*L. salivarius*	1.35	n/a	n/a
APTES-PDMA	n/a	*L. salivarius*	1.21	n/a	n/a

[a] Gao, G., et al. (2011). "The biocompatibility and biofilm resistance of implant coatings based on hydrophilic polymer brushes conjugated with antimicrobial peptides." Biomaterials 32(16): 3899-3909.

[b] Chen, R., et al. (2016). "Antimicrobial peptide melimine coating for titanium and its in vivo antibacterial activity in rodent subcutaneous infection models." Biomaterials 85: 142-151.

[c] Statz, A. R., et al. (2008). "Surface-immobilised antimicrobial peptoids." Biofouling 24(6): 439-448.

[d] Gabriel, M., et al. (2006). "Preparation of LL-37-grafted titanium surfaces with bactericidal activity." Bioconjugate chemistry 17(2): 548-550.

[e] Godoy-Gallardo, M., et al. (2014). "Covalent immobilization of hLf1-11 peptide on a titanium surface reduces bacterial adhesion and biofilm formation." Acta biomaterialia 10(8): 3522-3534.

[f] Godoy-Gallardo, M., et al. (2015). "Antibacterial properties of hLf1–11 peptide onto titanium surfaces: a comparison study between silanization and surface initiated polymerization." Biomacromolecules 16(2): 483-496.

2.7. Ellipsometry data.

2.7.1. FBS adsorption.

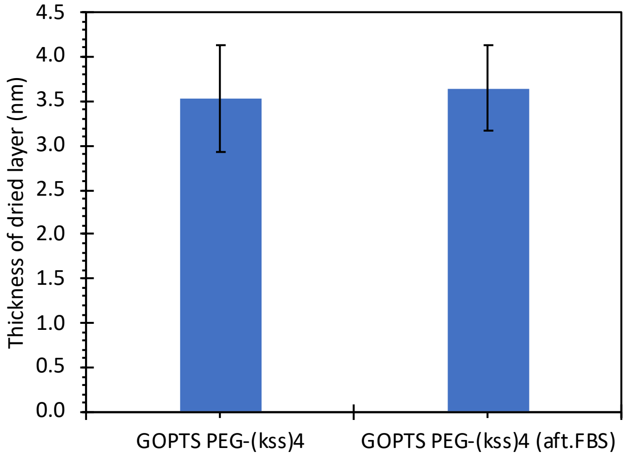

Figure S4. Ellipsometer dry layer thickness of independent samples measured before and after the FBS adsorption experiment (2 h incubation at r.t. in 10% fetal bovine serum prepared in 1X PBS). The thickness values were obtained from layer calculations made by the ellipsometer manufacturer's WVASE software (J.A. Woollam).

2.7.2. Lower bound estimation of $(kss)_4$ AMP separation.

Figure S4 shows that the entire GOPTS-PEG-N_3-$(kss)_4$ architecture has a total dry thickness of 3.5 nm as measured by ellipsometry. The molecular weight of this structure is ca. 4810 g/mole.[1] Using the same calculations as shown in sections 2.4.2 and 2.5, a chain density of 0.65 chain/nm^2 and an AMP separation of 3.9 nm is estimated.

This is an upper bound estimate in density (lower bound in separation) since, as explained in footnote 1, a significant portion of the measured thickness could be taken up by the GOPTS silane layer. We therefore use the 1.1 nm difference between this 3.9 nm estimate from ellipsometry and the 5 nm separation calculated from XPS data as the uncertainty in Fig. 5 of the main text, plotted as error bars. Even with such a generous bounding, the overall trend of increasing ratio of bacteria membrane disruption vs. attachment at increasing AMP separations

[1] This assumes that each PEG2k chain is associated with three GOPTS molecules (each 236 g/mol) and this configuration approximates an upper bound density of PEG. Each GOPTS has three reactive methoxy groups and the silane is expected to crosslink to form layers (e.g. 2~3 nm)[13] significantly thicker than a monolayer. Thus a significant part of the surface structure thickness measured actually belongs to the silane and not the PEG brush. (Silane monolayers can only be formed under especially well controlled conditions.) It is also not physically possible to attach a PEG2k chain to every GOPTS even if it were a silane monolayer because the polymer is much higher in molecular weight than GOPTS and has a larger areal footprint, and an immobilized PEG chain would hinder additional attachment around it (i.e. exhibit PEG's well-known anti-fouling effect).

is preserved in Fig. 5. Thus, our conclusion may be considered robust and is insensitive to the measurement uncertainties of either our XPS or ellipsometer results.

2.8. Comparison of live bacteria attachment on amino-silanes

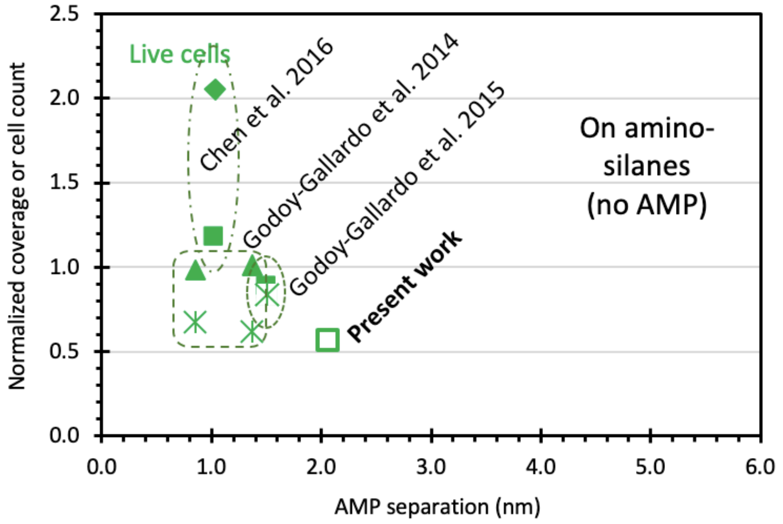

Figure S5. Live bacteria attachment on surfaces functionalized with amino-silanes, plotted against the average separation between the silane chains. The literature data is taken from Table S2. Open squares (□) indicate present study data for *P. aeruginosa*. Other symbols indicate *P. aeruginosa* (■),[14, 15] *S. aureus* (◆),[14] *L. salivarius* (▲),[16, 17] and *S. sanguinis* (✱).[16, 17]

3. References:

1. Zuckermann, R. N.; Kerr, J. M.; Kent, S. B.; Moos, W. H., Efficient method for the preparation of peptoids [oligo (N-substituted glycines)] by submonomer solid-phase synthesis. *Journal of the American Chemical Society* **1992,** *114* (26), 10646-10647.
2. Lau, K. H. A.; Sileika, T. S.; Park, S. H.; Sousa, A. M.; Burch, P.; Szleifer, I.; Messersmith, P. B., Molecular Design of Antifouling Polymer Brushes Using Sequence-Specific Peptoids. *Advanced materials interfaces* **2015,** *2* (1), 1400225.
3. Lau, K. H. A.; Castelletto, V.; Kendall, T.; Sefcik, J.; Hamley, I. W.; Reza, M.; Ruokolainen, J., Self-assembly of ultra-small micelles from amphiphilic lipopeptoids. *Chemical Communications* **2017,** *53* (13), 2178-2181.
4. Jonas, U.; Krüger, C., The effect of polar, nonpolar, and electrostatic interactions and wetting behavior on the particle assembly at patterned surfaces. *Journal of Supramolecular Chemistry* **2002,** *2* (1), 255-270.
5. Pop-Georgievski, O.; Popelka, S. t. p. n.; Houska, M.; Chvostová, D.; Proks, V.; Rypáček, F. e., Poly (ethylene oxide) layers grafted to dopamine-melanin anchoring layer: stability and resistance to protein adsorption. *Biomacromolecules* **2011,** *12* (9), 3232-3242.
6. Rostovtsev, V. V.; Green, L. G.; Fokin, V. V.; Sharpless, K. B., A stepwise huisgen cycloaddition process: copper (I)-catalyzed regioselective "ligation" of azides and terminal alkynes. *Angew. Chem. Int. Ed.* **2002,** *41* (14), 2596-2599.

7. Chapman, R.; Jolliffe, K. A.; Perrier, S., Synthesis of self-assembling cyclic peptide-polymer conjugates using click chemistry. *Aust. J. Chem.* **2010,** *63* (8), 1169-1172.
8. Meldal, M.; Tornøe, C. W., Cu-catalyzed azide− alkyne cycloaddition. *Chem. Rev.* **2008,** *108* (8), 2952-3015.
9. Hasan, A.; Saxena, V.; Pandey, L. M., Surface Functionalization of Ti6Al4V via Self-assembled Monolayers for Improved Protein Adsorption and Fibroblast Adhesion. *Langmuir* **2018,** *34* (11), 3494-3506.
10. Hasan, A.; Pattanayek, S. K.; Pandey, L. M., Effect of Functional Groups of Self-Assembled Monolayers on Protein Adsorption and Initial Cell Adhesion. *ACS Biomaterials Science & Engineering* **2018,** *4* (9), 3224-3233.
11. Lau, K. H. A.; Ren, C.; Park, S. H.; Szleifer, I.; Messersmith, P. B., An Experimental–Theoretical Analysis of Protein Adsorption on Peptidomimetic Polymer Brushes. *Langmuir* **2012,** *28* (4), 2288-2298.
12. Lau, K. H. A.; Ren, C.; Sileika, T. S.; Park, S. H.; Szleifer, I.; Messersmith, P. B., Surface-grafted polysarcosine as a peptoid antifouling polymer brush. *Langmuir* **2012,** *28* (46), 16099-16107.
13. Lau, K. H. A.; Duran, H.; Knoll, W., In situ Characterization of N-Carboxy Anhydride Polymerization in Nanoporous Anodic Alumina. *J. Phys. Chem. B* **2009,** *113* (10), 3179-3189.
14. Chen, R.; Willcox, M. D.; Ho, K. K. K.; Smyth, D.; Kumar, N., Antimicrobial peptide melimine coating for titanium and its in vivo antibacterial activity in rodent subcutaneous infection models. *Biomaterials* **2016,** *85*, 142-151.
15. Gao, G.; Lange, D.; Hilpert, K.; Kindrachuk, J.; Zou, Y.; Cheng, J. T. J.; Kazemzadeh-Narbat, M.; Yu, K.; Wang, R.; Straus, S. K.; Brooks, D. E.; Chew, B. H.; Hancock, R. E. W.; Kizhakkedathu, J. N., The biocompatibility and biofilm resistance of implant coatings based on hydrophilic polymer brushes conjugated with antimicrobial peptides. *Biomaterials* **2011,** *32* (16), 3899-3909.
16. Godoy-Gallardo, M.; Mas-Moruno, C.; Yu, K.; Manero, J. M.; Gil, F. J.; Kizhakkedathu, J. N.; Rodriguez, D., Antibacterial properties of hLf1–11 peptide onto titanium surfaces: a comparison study between silanization and surface initiated polymerization. *Biomacromolecules* **2015,** *16* (2), 483-496.
17. Godoy-Gallardo, M.; Mas-Moruno, C.; Fernández-Calderón, M. C.; Pérez-Giraldo, C.; Manero, J. M.; Albericio, F.; Gil, F. J.; Rodríguez, D., Covalent immobilization of hLf1-11 peptide on a titanium surface reduces bacterial adhesion and biofilm formation. *Acta Biomater.* **2014,** *10* (8), 3522-3534.
